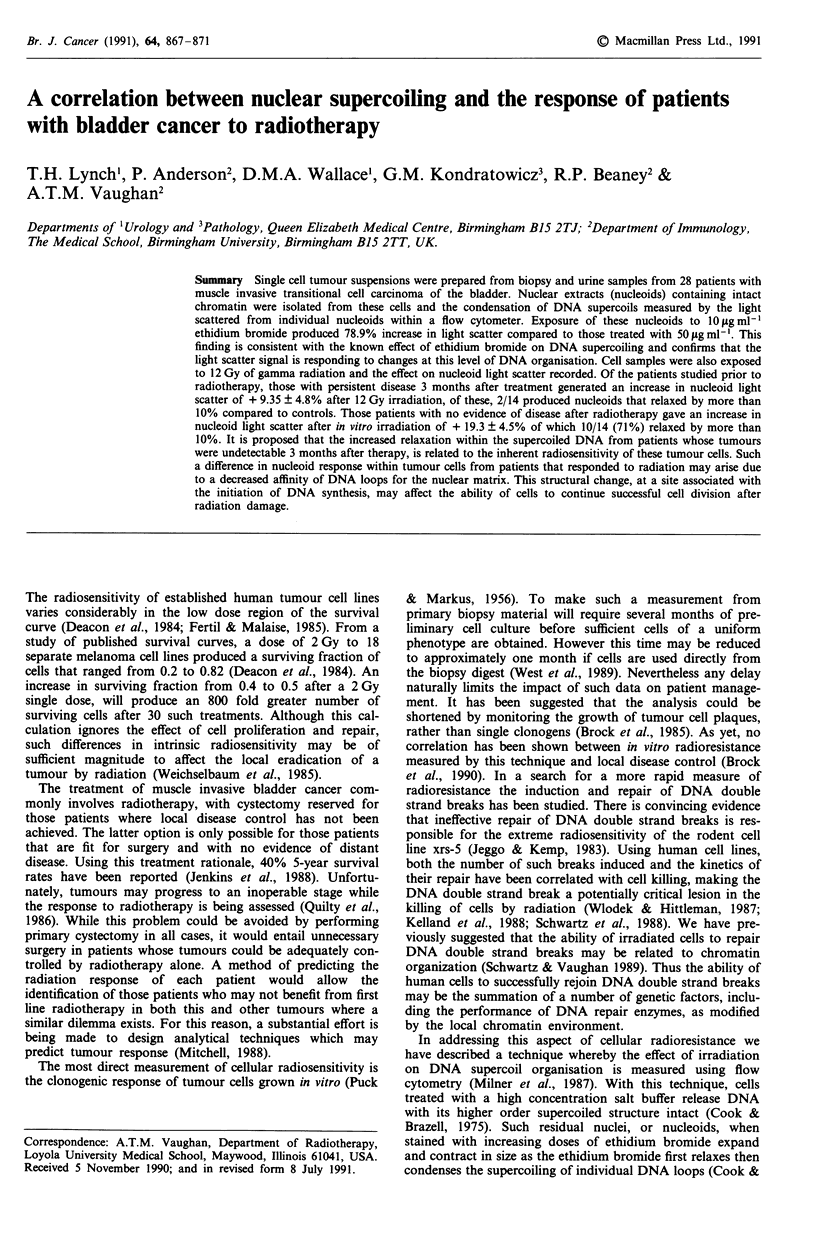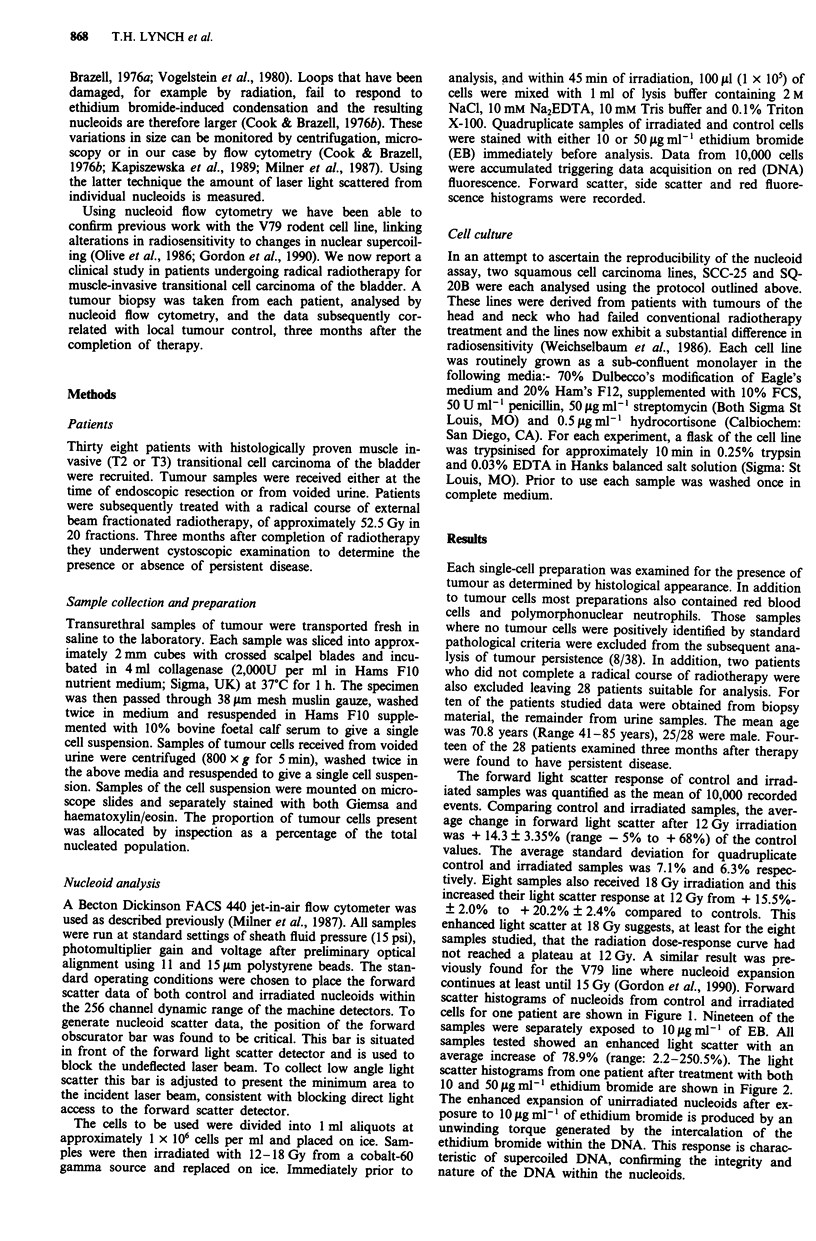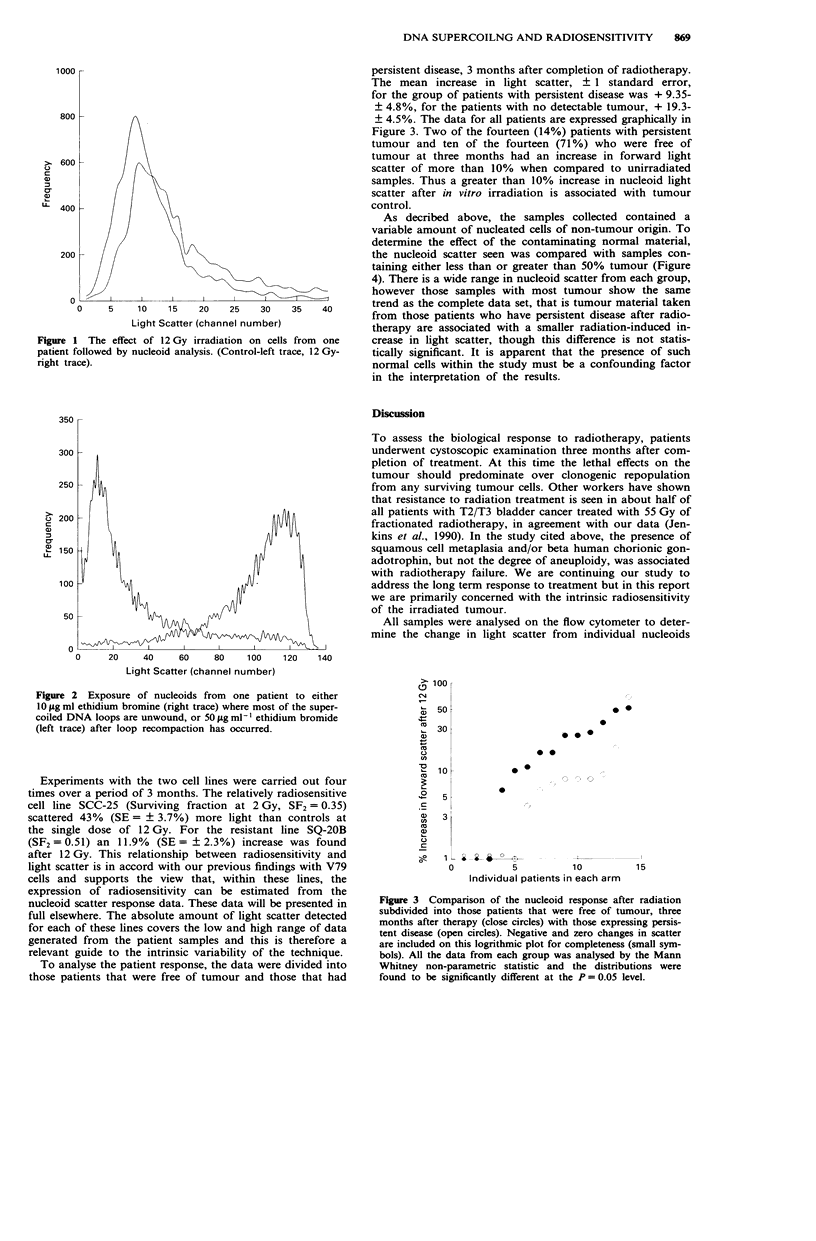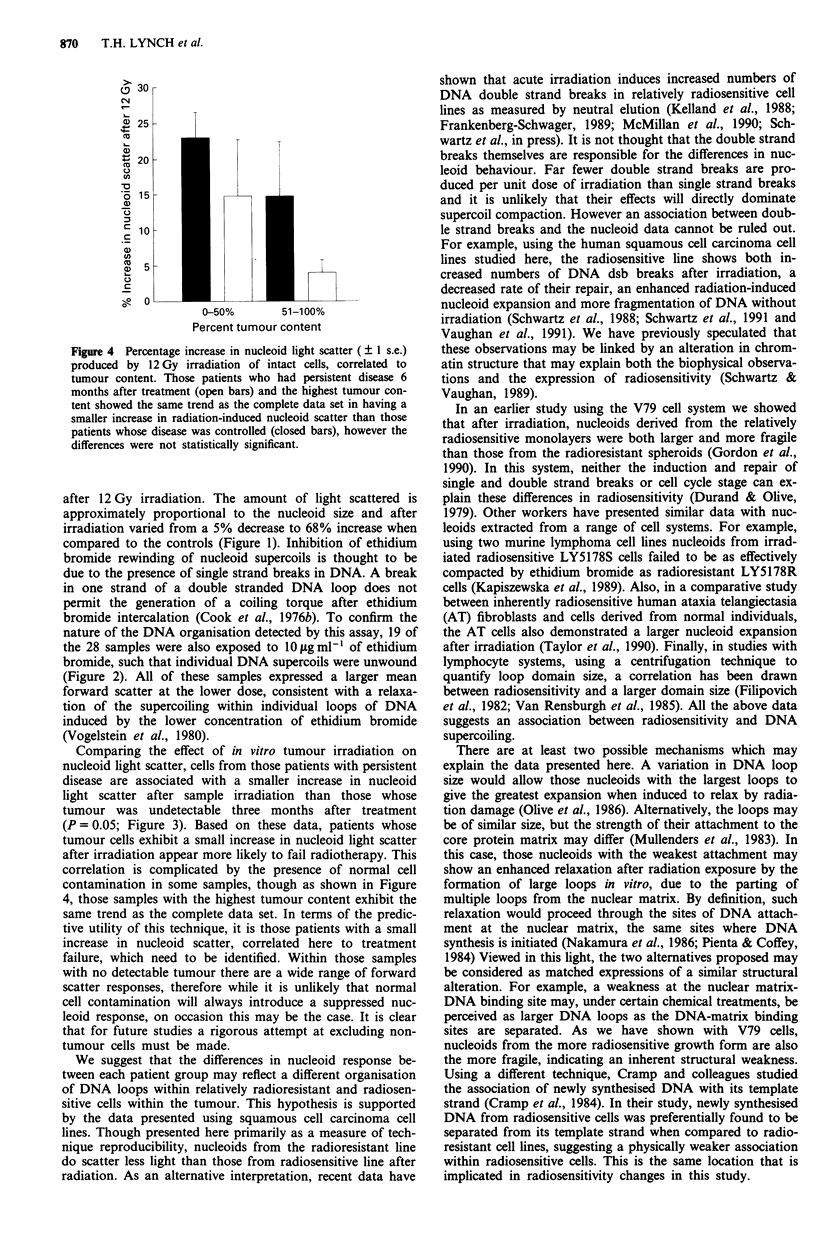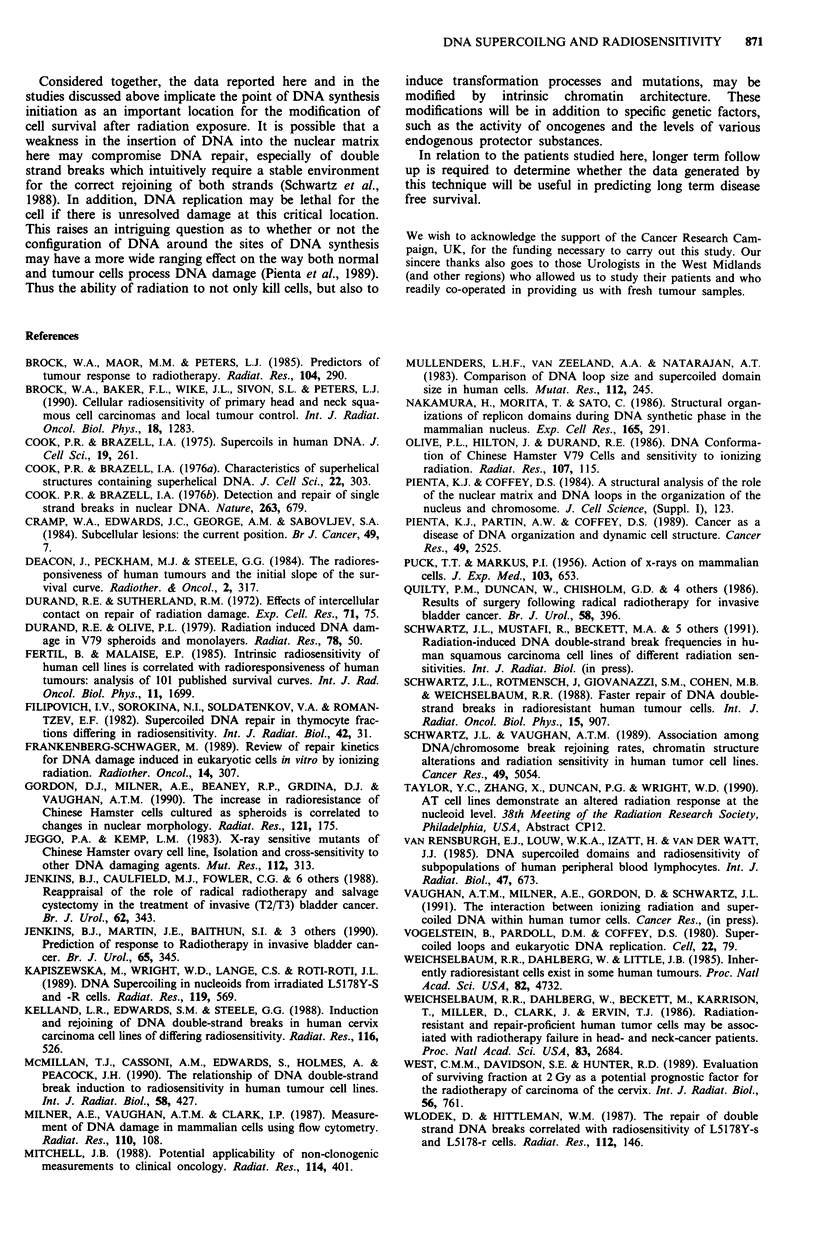# A correlation between nuclear supercoiling and the response of patients with bladder cancer to radiotherapy.

**DOI:** 10.1038/bjc.1991.415

**Published:** 1991-11

**Authors:** T. H. Lynch, P. Anderson, D. M. Wallace, G. M. Kondratowicz, R. P. Beaney, A. T. Vaughan

**Affiliations:** Department of Urology, Queen Elizabeth Medical Centre, Birmingham, UK.

## Abstract

Single cell tumour suspensions were prepared from biopsy and urine samples from 28 patients with muscle invasive transitional cell carcinoma of the bladder. Nuclear extracts (nucleoids) containing intact chromatin were isolated from these cells and the condensation of DNA supercoils measured by the light scattered from individual nucleoids within a flow cytometer. Exposure of these nucleoids to 10 micrograms ml-1 ethidium bromide produced 78.9% increase in light scatter compared to those treated with 50 micrograms ml-1. This finding is consistent with the known effect of ethidium bromide on DNA supercoiling and confirms that the light scatter signal is responding to changes at this level of DNA organisation. Cell samples were also exposed to 12 Gy of gamma radiation and the effect on nucleoid light scatter recorded. Of the patients studied prior to radiotherapy, those with persistent disease 3 months after treatment generated an increase in nucleoid light scatter of + 9.35 +/- 4.8% after 12 Gy irradiation, of these, 2/14 produced nucleoids that relaxed by more than 10% compared to controls. Those patients with no evidence of disease after radiotherapy gave an increase in nucleoid light scatter after in vitro irradiation of + 19.3 +/- 4.5% of which 10/14 (71%) relaxed by more than 10%. It is proposed that the increased relaxation within the supercoiled DNA from patients whose tumours were undetectable 3 months after therapy, is related to the inherent radiosensitivity of these tumour cells. Such a difference in nucleoid response within tumour cells from patients that responded to radiation may arise due to a decreased affinity of DNA loops for the nuclear matrix. This structural change, at a site associated with the initiation of DNA synthesis, may affect the ability of cells to continue successful cell division after radiation damage.


Br. J. Cancer (1991), 64, 867-871 © Macmillan Press Ltd., 1991

### A correlation between nuclear supercoiling and the response of patients with bladder cancer to radiotherapy

T.H. Lynch[1], P. Anderson[2], D.M.A. Wallace[1], G.M. Kondratowicz[3], R.P. Beaney[2] & A.T.M. Vaughan[2]

*Departments of [1]Urology and [3]Pathology, Queen Elizabeth Medical Centre, Birmingham B15 2TJ; [2]Department of Immunology, The Medical School, Birmingham University, Birmingham B15 2TT, UK.*

**Summary** Single cell tumour suspensions were prepared from biopsy and urine samples from 28 patients with muscle invasive transitional cell carcinoma of the bladder. Nuclear extracts (nucleoids) containing intact chromatin were isolated from these cells and the condensation of DNA supercoils measured by the light scattered from individual nucleoids within a flow cytometer. Exposure of these nucleoids to $10\,\mu g\,ml^{-1}$ ethidium bromide produced 78.9% increase in light scatter compared to those treated with $50\,\mu g\,ml^{-1}$. This finding is consistent with the known effect of ethidium bromide on DNA supercoiling and confirms that the light scatter signal is responding to changes at this level of DNA organisation. Cell samples were also exposed to 12 Gy of gamma radiation and the effect on nucleoid light scatter recorded. Of the patients studied prior to radiotherapy, those with persistent disease 3 months after treatment generated an increase in nucleoid light scatter of $+9.35 \pm 4.8\%$ after 12 Gy irradiation, of these, 2/14 produced nucleoids that relaxed by more than 10% compared to controls. Those patients with no evidence of disease after radiotherapy gave an increase in nucleoid light scatter after *in vitro* irradiation of $+19.3 \pm 4.5\%$ of which 10/14 (71%) relaxed by more than 10%. It is proposed that the increased relaxation within the supercoiled DNA from patients whose tumours were undetectable 3 months after therapy, is related to the inherent radiosensitivity of these tumour cells. Such a difference in nucleoid response within tumour cells from patients that responded to radiation may arise due to a decreased affinity of DNA loops for the nuclear matrix. This structural change, at a site associated with the initiation of DNA synthesis, may affect the ability of cells to continue successful cell division after radiation damage.

The radiosensitivity of established human tumour cell lines varies considerably in the low dose region of the survival curve (Deacon *et al.*, 1984; Fertil & Malaise, 1985). From a study of published survival curves, a dose of 2 Gy to 18 separate melanoma cell lines produced a surviving fraction of cells that ranged from 0.2 to 0.82 (Deacon *et al.*, 1984). An increase in surviving fraction from 0.4 to 0.5 after a 2 Gy single dose, will produce an 800 fold greater number of surviving cells after 30 such treatments. Although this calculation ignores the effect of cell proliferation and repair, such differences in intrinsic radiosensitivity may be of sufficient magnitude to affect the local eradication of a tumour by radiation (Weichselbaum *et al.*, 1985).

The treatment of muscle invasive bladder cancer commonly involves radiotherapy, with cystectomy reserved for those patients where local disease control has not been achieved. The latter option is only possible for those patients that are fit for surgery and with no evidence of distant disease. Using this treatment rationale, 40% 5-year survival rates have been reported (Jenkins *et al.*, 1988). Unfortunately, tumours may progress to an inoperable stage while the response to radiotherapy is being assessed (Quilty *et al.*, 1986). While this problem could be avoided by performing primary cystectomy in all cases, it would entail unnecessary surgery in patients whose tumours could be adequately controlled by radiotherapy alone. A method of predicting the radiation response of each patient would allow the identification of those patients who may not benefit from first line radiotherapy in both this and other tumours where a similar dilemma exists. For this reason, a substantial effort is being made to design analytical techniques which may predict tumour response (Mitchell, 1988).

The most direct measurement of cellular radiosensitivity is the clonogenic response of tumour cells grown *in vitro* (Puck & Markus, 1956). To make such a measurement from primary biopsy material will require several months of preliminary cell culture before sufficient cells of a uniform phenotype are obtained. However this time may be reduced to approximately one month if cells are used directly from the biopsy digest (West *et al.*, 1989). Nevertheless any delay naturally limits the impact of such data on patient management. It has been suggested that the analysis could be shortened by monitoring the growth of tumour cell plaques, rather than single clonogens (Brock *et al.*, 1985). As yet, no correlation has been shown between *in vitro* radioresistance measured by this technique and local disease control (Brock *et al.*, 1990). In a search for a more rapid measure of radioresistance the induction and repair of DNA double strand breaks has been studied. There is convincing evidence that ineffective repair of DNA double strand breaks is responsible for the extreme radiosensitivity of the rodent cell line xrs-5 (Jeggo & Kemp, 1983). Using human cell lines, both the number of such breaks induced and the kinetics of their repair have been correlated with cell killing, making the DNA double strand break a potentially critical lesion in the killing of cells by radiation (Wlodek & Hittleman, 1987; Kelland *et al.*, 1988; Schwartz *et al.*, 1988). We have previously suggested that the ability of irradiated cells to repair DNA double strand breaks may be related to chromatin organization (Schwartz & Vaughan 1989). Thus the ability of human cells to successfully rejoin DNA double strand breaks may be the summation of a number of genetic factors, including the performance of DNA repair enzymes, as modified by the local chromatin environment.

In addressing this aspect of cellular radioresistance we have described a technique whereby the effect of irradiation on DNA supercoil organisation is measured using flow cytometry (Milner *et al.*, 1987). With this technique, cells treated with a high concentration salt buffer release DNA with its higher order supercoiled structure intact (Cook & Brazell, 1975). Such residual nuclei, or nucleoids, when stained with increasing doses of ethidium bromide expand and contract in size as the ethidium bromide first relaxes then condenses the supercoiling of individual DNA loops (Cook &

Correspondence: A.T.M. Vaughan, Department of Radiotherapy, Loyola University Medical School, Maywood, Illinois 61041, USA.
Received 5 November 1990; and in revised form 8 July 1991.

Brazell, 1976a; Vogelstein et al., 1980). Loops that have been damaged, for example by radiation, fail to respond to ethidium bromide-induced condensation and the resulting nucleoids are therefore larger (Cook & Brazell, 1976b). These variations in size can be monitored by centrifugation, microscopy or in our case by flow cytometry (Cook & Brazell, 1976b; Kapiszewska et al., 1989; Milner et al., 1987). Using the latter technique the amount of laser light scattered from individual nucleoids is measured.

Using nucleoid flow cytometry we have been able to confirm previous work with the V79 rodent cell line, linking alterations in radiosensitivity to changes in nuclear supercoiling (Olive et al., 1986; Gordon et al., 1990). We now report a clinical study in patients undergoing radical radiotherapy for muscle-invasive transitional cell carcinoma of the bladder. A tumour biopsy was taken from each patient, analysed by nucleoid flow cytometry, and the data subsequently correlated with local tumour control, three months after the completion of therapy.

### Methods

### *Patients*

Thirty eight patients with histologically proven muscle invasive (T2 or T3) transitional cell carcinoma of the bladder were recruited. Tumour samples were received either at the time of endoscopic resection or from voided urine. Patients were subsequently treated with a radical course of external beam fractionated radiotherapy, of approximately 52.5 Gy in 20 fractions. Three months after completion of radiotherapy they underwent cystoscopic examination to determine the presence or absence of persistent disease.

### *Sample collection and preparation*

Transurethral samples of tumour were transported fresh in saline to the laboratory. Each sample was sliced into approximately 2 mm cubes with crossed scalpel blades and incubated in 4 ml collagenase (2,000U per ml in Hams F10 nutrient medium; Sigma, UK) at 37°C for 1 h. The specimen was then passed through 38 $\mu$m mesh muslin gauze, washed twice in medium and resuspended in Hams F10 supplemented with 10% bovine foetal calf serum to give a single cell suspension. Samples of tumour cells received from voided urine were centrifuged (800 $\times g$ for 5 min), washed twice in the above media and resuspended to give a single cell suspension. Samples of the cell suspension were mounted on microscope slides and separately stained with both Giemsa and haematoxylin/eosin. The proportion of tumour cells present was allocated by inspection as a percentage of the total nucleated population.

### *Nucleoid analysis*

A Becton Dickinson FACS 440 jet-in-air flow cytometer was used as described previously (Milner et al., 1987). All samples were run at standard settings of sheath fluid pressure (15 psi), photomultiplier gain and voltage after preliminary optical alignment using 11 and 15 $\mu$m polystyrene beads. The standard operating conditions were chosen to place the forward scatter data of both control and irradiated nucleoids within the 256 channel dynamic range of the machine detectors. To generate nucleoid scatter data, the position of the forward obscurator bar was found to be critical. This bar is situated in front of the forward light scatter detector and is used to block the undeflected laser beam. To collect low angle light scatter this bar is adjusted to present the minimum area to the incident laser beam, consistent with blocking direct light access to the forward scatter detector.

The cells to be used were divided into 1 ml aliquots at approximately $1 \times 10^6$ cells per ml and placed on ice. Samples were then irradiated with 12–18 Gy from a cobalt-60 gamma source and replaced on ice. Immediately prior to analysis, and within 45 min of irradiation, 100 $\mu$l ($1 \times 10^5$) of cells were mixed with 1 ml of lysis buffer containing 2 M NaCl, 10 mM $Na_2EDTA$, 10 mM Tris buffer and 0.1% Triton X-100. Quadruplicate samples of irradiated and control cells were stained with either 10 or 50 $\mu g\,ml^{-1}$ ethidium bromide (EB) immediately before analysis. Data from 10,000 cells were accumulated triggering data acquisition on red (DNA) fluorescence. Forward scatter, side scatter and red fluorescence histograms were recorded.

### *Cell culture*

In an attempt to ascertain the reproducibility of the nucleoid assay, two squamous cell carcinoma lines, SCC-25 and SQ-20B were each analysed using the protocol outlined above. These lines were derived from patients with tumours of the head and neck who had failed conventional radiotherapy treatment and the lines now exhibit a substantial difference in radiosensitivity (Weichselbaum et al., 1986). Each cell line was routinely grown as a sub-confluent monolayer in the following media:- 70% Dulbecco's modification of Eagle's medium and 20% Ham's F12, supplemented with 10% FCS, 50 U $ml^{-1}$ penicillin, 50 $\mu g\,ml^{-1}$ streptomycin (Both Sigma St Louis, MO) and 0.5 $\mu g\,ml^{-1}$ hydrocortisone (Calbiochem: San Diego, CA). For each experiment, a flask of the cell line was trypsinised for approximately 10 min in 0.25% trypsin and 0.03% EDTA in Hanks balanced salt solution (Sigma: St Louis, MO). Prior to use each sample was washed once in complete medium.

### Results

Each single-cell preparation was examined for the presence of tumour as determined by histological appearance. In addition to tumour cells most preparations also contained red blood cells and polymorphonuclear neutrophils. Those samples where no tumour cells were positively identified by standard pathological criteria were excluded from the subsequent analysis of tumour persistence (8/38). In addition, two patients who did not complete a radical course of radiotherapy were also excluded leaving 28 patients suitable for analysis. For ten of the patients studied data were obtained from biopsy material, the remainder from urine samples. The mean age was 70.8 years (Range 41–85 years), 25/28 were male. Fourteen of the 28 patients examined three months after therapy were found to have persistent disease.

The forward light scatter response of control and irradiated samples was quantified as the mean of 10,000 recorded events. Comparing control and irradiated samples, the average change in forward light scatter after 12 Gy irradiation was + 14.3 ± 3.35% (range – 5% to + 68%) of the control values. The average standard deviation for quadruplicate control and irradiated samples was 7.1% and 6.3% respectively. Eight samples also received 18 Gy irradiation and this increased their light scatter response at 12 Gy from + 15.5%- ± 2.0% to + 20.2% ± 2.4% compared to controls. This enhanced light scatter at 18 Gy suggests, at least for the eight samples studied, that the radiation dose-response curve had not reached a plateau at 12 Gy. A similar result was previously found for the V79 line where nucleoid expansion continues at least until 15 Gy (Gordon et al., 1990). Forward scatter histograms of nucleoids from control and irradiated cells for one patient are shown in Figure 1. Nineteen of the samples were separately exposed to 10 $\mu g\,ml^{-1}$ of EB. All samples tested showed an enhanced light scatter with an average increase of 78.9% (range: 2.2–250.5%). The light scatter histograms from one patient after treatment with both 10 and 50 $\mu g\,ml^{-1}$ ethidium bromide are shown in Figure 2. The enhanced expansion of unirradiated nucleoids after exposure to 10 $\mu g\,ml^{-1}$ of ethidium bromide is produced by an unwinding torque generated by the intercalation of the ethidium bromide within the DNA. This response is characteristic of supercoiled DNA, confirming the integrity and nature of the DNA within the nucleoids.

**Figure 1** The effect of 12 Gy irradiation on cells from one patient followed by nucleoid analysis. (Control-left trace, 12 Gy-right trace).

**Figure 2** Exposure of nucleoids from one patient to either 10 μg ml ethidium bromine (right trace) where most of the supercoiled DNA loops are unwound, or 50 μg ml$^{-1}$ ethidium bromide (left trace) after loop recompaction has occurred.

Experiments with the two cell lines were carried out four times over a period of 3 months. The relatively radiosensitive cell line SCC-25 (Surviving fraction at 2 Gy, $SF_2 = 0.35$) scattered 43% (SE = ± 3.7%) more light than controls at the single dose of 12 Gy. For the resistant line SQ-20B ($SF_2 = 0.51$) an 11.9% (SE = ± 2.3%) increase was found after 12 Gy. This relationship between radiosensitivity and light scatter is in accord with our previous findings with V79 cells and supports the view that, within these lines, the expression of radiosensitivity can be estimated from the nucleoid scatter response data. These data will be presented in full elsewhere. The absolute amount of light scatter detected for each of these lines covers the low and high range of data generated from the patient samples and this is therefore a relevant guide to the intrinsic variability of the technique.

To analyse the patient response, the data were divided into those patients that were free of tumour and those that had persistent disease, 3 months after completion of radiotherapy. The mean increase in light scatter, ± 1 standard error, for the group of patients with persistent disease was + 9.35- ± 4.8%, for the patients with no detectable tumour, + 19.3- ± 4.5%. The data for all patients are expressed graphically in Figure 3. Two of the fourteen (14%) patients with persistent tumour and ten of the fourteen (71%) who were free of tumour at three months had an increase in forward light scatter of more than 10% when compared to unirradiated samples. Thus a greater than 10% increase in nucleoid light scatter after *in vitro* irradiation is associated with tumour control.

As decribed above, the samples collected contained a variable amount of nucleated cells of non-tumour origin. To determine the effect of the contaminating normal material, the nucleoid scatter seen was compared with samples containing either less than or greater than 50% tumour (Figure 4). There is a wide range in nucleoid scatter from each group, however those samples with most tumour show the same trend as the complete data set, that is tumour material taken from those patients who have persistent disease after radiotherapy are associated with a smaller radiation-induced increase in light scatter, though this difference is not statistically significant. It is apparent that the presence of such normal cells within the study must be a confounding factor in the interpretation of the results.

### Discussion

To assess the biological response to radiotherapy, patients underwent cystoscopic examination three months after completion of treatment. At this time the lethal effects on the tumour should predominate over clonogenic repopulation from any surviving tumour cells. Other workers have shown that resistance to radiation treatment is seen in about half of all patients with T2/T3 bladder cancer treated with 55 Gy of fractionated radiotherapy, in agreement with our data (Jenkins *et al.*, 1990). In the study cited above, the presence of squamous cell metaplasia and/or beta human chorionic gonadotrophin, but not the degree of aneuploidy, was associated with radiotherapy failure. We are continuing our study to address the long term response to treatment but in this report we are primarily concerned with the intrinsic radiosensitivity of the irradiated tumour.

All samples were analysed on the flow cytometer to determine the change in light scatter from individual nucleoids

**Figure 3** Comparison of the nucleoid response after radiation subdivided into those patients that were free of tumour, three months after therapy (close circles) with those expressing persistent disease (open circles). Negative and zero changes in scatter are included on this logrithmic plot for completeness (small symbols). All the data from each group was analysed by the Mann Whitney non-parametric statistic and the distributions were found to be significantly different at the $P = 0.05$ level.

**Figure 4** Percentage increase in nucleoid light scatter (± 1 s.e.) produced by 12 Gy irradiation of intact cells, correlated to tumour content. Those patients who had persistent disease 6 months after treatment (open bars) and the highest tumour content showed the same trend as the complete data set in having a smaller increase in radiation-induced nucleoid scatter than those patients whose disease was controlled (closed bars), however the differences were not statistically significant.

after 12 Gy irradiation. The amount of light scattered is approximately proportional to the nucleoid size and after irradiation varied from a 5% decrease to 68% increase when compared to the controls (Figure 1). Inhibition of ethidium bromide rewinding of nucleoid supercoils is thought to be due to the presence of single strand breaks in DNA. A break in one strand of a double stranded DNA loop does not permit the generation of a coiling torque after ethidium bromide intercalation (Cook *et al.*, 1976*b*). To confirm the nature of the DNA organisation detected by this assay, 19 of the 28 samples were also exposed to 10 $\mu g\, ml^{-1}$ of ethidium bromide, such that individual DNA supercoils were unwound (Figure 2). All of these samples expressed a larger mean forward scatter at the lower dose, consistent with a relaxation of the supercoiling within individual loops of DNA induced by the lower concentration of ethidium bromide (Vogelstein *et al.*, 1980).

Comparing the effect of *in vitro* tumour irradiation on nucleoid light scatter, cells from those patients with persistent disease are associated with a smaller increase in nucleoid light scatter after sample irradiation than those whose tumour was undetectable three months after treatment ($P = 0.05$; Figure 3). Based on these data, patients whose tumour cells exhibit a small increase in nucleoid light scatter after irradiation appear more likely to fail radiotherapy. This correlation is complicated by the presence of normal cell contamination in some samples, though as shown in Figure 4, those samples with the highest tumour content exhibit the same trend as the complete data set. In terms of the predictive utility of this technique, it is those patients with a small increase in nucleoid scatter, correlated here to treatment failure, which need to be identified. Within those samples with no detectable tumour there are a wide range of forward scatter responses, therefore while it is unlikely that normal cell contamination will always introduce a suppressed nucleoid response, on occasion this may be the case. It is clear that for future studies a rigorous attempt at excluding non-tumour cells must be made.

We suggest that the differences in nucleoid response between each patient group may reflect a different organisation of DNA loops within relatively radioresistant and radiosensitive cells within the tumour. This hypothesis is supported by the data presented using squamous cell carcinoma cell lines. Though presented here primarily as a measure of technique reproducibility, nucleoids from the radioresistant line do scatter less light than those from radiosensitive line after radiation. As an alternative interpretation, recent data have shown that acute irradiation induces increased numbers of DNA double strand breaks in relatively radiosensitive cell lines as measured by neutral elution (Kelland *et al.*, 1988; Frankenberg-Schwager, 1989; McMillan *et al.*, 1990; Schwartz *et al.*, in press). It is not thought that the double strand breaks themselves are responsible for the differences in nucleoid behaviour. Far fewer double strand breaks are produced per unit dose of irradiation than single strand breaks and it is unlikely that their effects will directly dominate supercoil compaction. However an association between double strand breaks and the nucleoid data cannot be ruled out. For example, using the human squamous cell carcinoma cell lines studied here, the radiosensitive line shows both increased numbers of DNA dsb breaks after irradiation, a decreased rate of their repair, an enhanced radiation-induced nucleoid expansion and more fragmentation of DNA without irradiation (Schwartz *et al.*, 1988; Schwartz *et al.*, 1991 and Vaughan *et al.*, 1991). We have previously speculated that these observations may be linked by an alteration in chromatin structure that may explain both the biophysical observations and the expression of radiosensitivity (Schwartz & Vaughan, 1989).

In an earlier study using the V79 cell system we showed that after irradiation, nucleoids derived from the relatively radiosensitive monolayers were both larger and more fragile than those from the radioresistant spheroids (Gordon *et al.*, 1990). In this system, neither the induction and repair of single and double strand breaks or cell cycle stage can explain these differences in radiosensitivity (Durand & Olive, 1979). Other workers have presented similar data with nucleoids extracted from a range of cell systems. For example, using two murine lymphoma cell lines nucleoids from irradiated radiosensitive LY5178S cells failed to be as effectively compacted by ethidium bromide as radioresistant LY5178R cells (Kapiszewska *et al.*, 1989). Also, in a comparative study between inherently radiosensitive human ataxia telangiectasia (AT) fibroblasts and cells derived from normal individuals, the AT cells also demonstrated a larger nucleoid expansion after irradiation (Taylor *et al.*, 1990). Finally, in studies with lymphocyte systems, using a centrifugation technique to quantify loop domain size, a correlation has been drawn between radiosensitivity and a larger domain size (Filipovich *et al.*, 1982; Van Rensburgh *et al.*, 1985). All the above data suggests an association between radiosensitivity and DNA supercoiling.

There are at least two possible mechanisms which may explain the data presented here. A variation in DNA loop size would allow those nucleoids with the largest loops to give the greatest expansion when induced to relax by radiation damage (Olive *et al.*, 1986). Alternatively, the loops may be of similar size, but the strength of their attachment to the core protein matrix may differ (Mullenders *et al.*, 1983). In this case, those nucleoids with the weakest attachment may show an enhanced relaxation after radiation exposure by the formation of large loops *in vitro*, due to the parting of multiple loops from the nuclear matrix. By definition, such relaxation would proceed through the sites of DNA attachment at the nuclear matrix, the same sites where DNA synthesis is initiated (Nakamura *et al.*, 1986; Pienta & Coffey, 1984) Viewed in this light, the two alternatives proposed may be considered as matched expressions of a similar structural alteration. For example, a weakness at the nuclear matrix-DNA binding site may, under certain chemical treatments, be perceived as larger DNA loops as the DNA-matrix binding sites are separated. As we have shown with V79 cells, nucleoids from the more radiosensitive growth form are also the more fragile, indicating an inherent structural weakness. Using a different technique, Cramp and colleagues studied the association of newly synthesised DNA with its template strand (Cramp *et al.*, 1984). In their study, newly synthesised DNA from radiosensitive cells was preferentially found to be separated from its template strand when compared to radioresistant cell lines, suggesting a physically weaker association within radiosensitive cells. This is the same location that is implicated in radiosensitivity changes in this study.

Considered together, the data reported here and in the studies discussed above implicate the point of DNA synthesis initiation as an important location for the modification of cell survival after radiation exposure. It is possible that a weakness in the insertion of DNA into the nuclear matrix here may compromise DNA repair, especially of double strand breaks which intuitively require a stable environment for the correct rejoining of both strands (Schwartz *et al.*, 1988). In addition, DNA replication may be lethal for the cell if there is unresolved damage at this critical location. This raises an intriguing question as to whether or not the configuration of DNA around the sites of DNA synthesis may have a more wide ranging effect on the way both normal and tumour cells process DNA damage (Pienta *et al.*, 1989). Thus the ability of radiation to not only kill cells, but also to induce transformation processes and mutations, may be modified by intrinsic chromatin architecture. These modifications will be in addition to specific genetic factors, such as the activity of oncogenes and the levels of various endogenous protector substances.

In relation to the patients studied here, longer term follow up is required to determine whether the data generated by this technique will be useful in predicting long term disease free survival.

We wish to acknowledge the support of the Cancer Research Campaign, UK, for the funding necessary to carry out this study. Our sincere thanks also goes to those Urologists in the West Midlands (and other regions) who allowed us to study their patients and who readily co-operated in providing us with fresh tumour samples.

### References

BROCK, W.A., MAOR, M.M. & PETERS, L.J. (1985). Predictors of tumour response to radiotherapy. *Radiat. Res.*, **104**, 290.

BROCK, W.A., BAKER, F.L., WIKE, J.L., SIVON, S.L. & PETERS, L.J. (1990). Cellular radiosensitivity of primary head and neck squamous cell carcinomas and local tumour control. *Int. J. Radiat. Oncol. Biol. Phys.*, **18**, 1283.

COOK, P.R. & BRAZELL, I.A. (1975). Supercoils in human DNA. *J. Cell Sci.*, **19**, 261.

COOK, P.R. & BRAZELL, I.A. (1976*a*). Characteristics of superhelical structures containing superhelical DNA. *J. Cell Sci.*, **22**, 303.

COOK. P.R. & BRAZELL, I.A. (1976*b*). Detection and repair of single strand breaks in nuclear DNA. *Nature*, **263**, 679.

CRAMP, W.A., EDWARDS, J.C., GEORGE, A.M. & SABOVLJEV, S.A. (1984). Subcellular lesions: the current position. *Br J. Cancer*, **49**, 7.

DEACON, J., PECKHAM, M.J. & STEELE, G.G. (1984). The radioresponsiveness of human tumours and the initial slope of the survival curve. *Radiother. & Oncol.*, **2**, 317.

DURAND, R.E. & SUTHERLAND, R.M. (1972). Effects of intercellular contact on repair of radiation damage. *Exp. Cell. Res.*, **71**, 75.

DURAND, R.E. & OLIVE, P.L. (1979). Radiation induced DNA damage in V79 spheroids and monolayers. *Radiat. Res.*, **78**, 50.

FERTIL, B. & MALAISE, E.P. (1985). Intrinsic radiosensitivity of human cell lines is correlated with radioresponsiveness of human tumours: analysis of 101 published survival curves. *Int. J. Rad. Oncol. Biol. Phys.*, **11**, 1699.

FILIPOVICH, I.V., SOROKINA, N.I., SOLDATENKOV, V.A. & ROMANTZEV, E.F. (1982). Supercoiled DNA repair in thymocyte fractions differing in radiosensitivity. *Int. J. Radiat. Biol.*, **42**, 31.

FRANKENBERG-SCHWAGER, M. (1989). Review of repair kinetics for DNA damage induced in eukaryotic cells *in vitro* by ionizing radiation. *Radiother. Oncol.*, **14**, 307.

GORDON, D.J., MILNER, A.E., BEANEY, R.P., GRDINA, D.J. & VAUGHAN, A.T.M. (1990). The increase in radioresistance of Chinese Hamster cells cultured as spheroids is correlated to changes in nuclear morphology. *Radiat. Res.*, **121**, 175.

JEGGO, P.A. & KEMP, L.M. (1983). X-ray sensitive mutants of Chinese Hamster ovary cell line, Isolation and cross-sensitivity to other DNA damaging agents. *Mut. Res.*, **112**, 313.

JENKINS, B.J., CAULFIELD, M.J., FOWLER, C.G. & 6 others (1988). Reappraisal of the role of radical radiotherapy and salvage cystectomy in the treatment of invasive (T2/T3) bladder cancer. *Br. J. Urol.*, **62**, 343.

JENKINS, B.J., MARTIN, J.E., BAITHUN, S.I. & 3 others (1990). Prediction of response to Radiotherapy in invasive bladder cancer. *Br. J. Urol.*, **65**, 345.

KAPISZEWSKA, M., WRIGHT, W.D., LANGE, C.S. & ROTI-ROTI, J.L. (1989). DNA Supercoiling in nucleoids from irradiated L5178Y-S and -R cells. *Radiat. Res.*, **119**, 569.

KELLAND, L.R., EDWARDS, S.M. & STEELE, G.G. (1988). Induction and rejoining of DNA double-strand breaks in human cervix carcinoma cell lines of differing radiosensitivity. *Radiat. Res.*, **116**, 526.

MCMILLAN, T.J., CASSONI, A.M., EDWARDS, S., HOLMES, A. & PEACOCK, J.H. (1990). The relationship of DNA double-strand break induction to radiosensitivity in human tumour cell lines. *Int. J. Radiat. Biol.*, **58**, 427.

MILNER, A.E., VAUGHAN, A.T.M. & CLARK, I.P. (1987). Measurement of DNA damage in mammalian cells using flow cytometry. *Radiat. Res.*, **110**, 108.

MITCHELL, J.B. (1988). Potential applicability of non-clonogenic measurements to clinical oncology. *Radiat. Res.*, **114**, 401.

MULLENDERS, L.H.F., VAN ZEELAND, A.A. & NATARAJAN, A.T. (1983). Comparison of DNA loop size and supercoiled domain size in human cells. *Mutat. Res.*, **112**, 245.

NAKAMURA, H., MORITA, T. & SATO, C. (1986). Structural organizations of replicon domains during DNA synthetic phase in the mammalian nucleus. *Exp. Cell Res.*, **165**, 291.

OLIVE, P.L., HILTON, J. & DURAND, R.E. (1986). DNA Conformation of Chinese Hamster V79 Cells and sensitivity to ionizing radiation. *Radiat. Res.*, **107**, 115.

PIENTA, K.J. & COFFEY, D.S. (1984). A structural analysis of the role of the nuclear matrix and DNA loops in the organization of the nucleus and chromosome. *J. Cell Science*, (Suppl. I), 123.

PIENTA, K.J., PARTIN, A.W. & COFFEY, D.S. (1989). Cancer as a disease of DNA organization and dynamic cell structure. *Cancer Res.*, **49**, 2525.

PUCK, T.T. & MARKUS, P.I. (1956). Action of x-rays on mammalian cells. *J. Exp. Med.*, **103**, 653.

QUILTY, P.M., DUNCAN, W., CHISHOLM, G.D. & 4 others (1986). Results of surgery following radical radiotherapy for invasive bladder cancer. *Br. J. Urol.*, **58**, 396.

SCHWARTZ, J.L., MUSTAFI, R., BECKETT, M.A. & 5 others (1991). Radiation-induced DNA double-strand break frequencies in human squamous carcinoma cell lines of different radiation sensitivities. *Int. J. Radiat. Biol.* (in press).

SCHWARTZ, J.L., ROTMENSCH, J, GIOVANAZZI, S.M., COHEN, M.B. & WEICHSELBAUM, R.R. (1988). Faster repair of DNA double-strand breaks in radioresistant human tumour cells. *Int. J. Radiat. Oncol. Biol. Phys.*, **15**, 907.

SCHWARTZ, J.L. & VAUGHAN, A.T.M. (1989). Association among DNA/chromosome break rejoining rates, chromatin structure alterations and radiation sensitivity in human tumor cell lines. *Cancer Res.*, **49**, 5054.

TAYLOR, Y.C., ZHANG, X., DUNCAN, P.G. & WRIGHT, W.D. (1990). AT cell lines demonstrate an altered radiation response at the nucleoid level. *38th Meeting of the Radiation Research Society, Philadelphia, USA*, Abstract CP12.

VAN RENSBURGH, E.J., LOUW, W.K.A., IZATT, H. & VAN DER WATT, J.J. (1985). DNA supercoiled domains and radiosensitivity of subpopulations of human peripheral blood lymphocytes. *Int. J. Radiat. Biol.*, **47**, 673.

VAUGHAN, A.T.M., MILNER, A.E., GORDON, D. & SCHWARTZ, J.L. (1991). The interaction between ionizing radiation and supercoiled DNA within human tumor cells. *Cancer Res.*, (in press).

VOGELSTEIN, B., PARDOLL, D.M. & COFFEY, D.S. (1980). Supercoiled loops and eukaryotic DNA replication. *Cell*, **22**, 79.

WEICHSELBAUM, R.R., DAHLBERG, W. & LITTLE, J.B. (1985). Inherently radioresistant cells exist in some human tumours. *Proc. Natl Acad. Sci. USA*, **82**, 4732.

WEICHSELBAUM, R.R., DAHLBERG, W., BECKETT, M., KARRISON, T., MILLER, D., CLARK, J. & ERVIN, T.J. (1986). Radiation-resistant and repair-proficient human tumor cells may be associated with radiotherapy failure in head- and neck-cancer patients. *Proc. Natl Acad. Sci. USA*, **83**, 2684.

WEST, C.M.M., DAVIDSON, S.E. & HUNTER, R.D. (1989). Evaluation of surviving fraction at 2 Gy as a potential prognostic factor for the radiotherapy of carcinoma of the cervix. *Int. J. Radiat. Biol.*, **56**, 761.

WLODEK, D. & HITTLEMAN, W.M. (1987). The repair of double strand DNA breaks correlated with radiosensitivity of L5178Y-s and L5178-r cells. *Radiat. Res.*, **112**, 146.